# Perception and satisfaction of cervical cancer screening by Visual Inspection with Acetic acid (VIA) at Meknes-Tafilalet Region, Morocco: a population-based cross-sectional study

**DOI:** 10.1186/s12905-015-0268-0

**Published:** 2015-11-24

**Authors:** Farida Selmouni, Ahmed Zidouh, Consuelo Alvarez-Plaza, Karima El Rhazi

**Affiliations:** Complutense University of Madrid, Madrid, Spain; Higher Institute of Nursing Professions and Techniques of Health of Rabat, Rabat, Morocco; Lalla Salma Foundation, Cancer Prevention and Treatment, Rabat, Morocco; Laboratory of Epidemiology, Clinical Research and Community Health, Sidi Mohamed Ben AbdIllah University, Fez, Morocco

**Keywords:** Cervical cancer, Visual inspection with acetic acid, Early detection, Perception, Satisfaction, Morocco

## Abstract

**Background:**

This study aims to explore the perception and satisfaction of cervical cancer screening by Visual Inspection with Acetic acid (VIA) in Meknes-Tafilalet Region among target women.

**Methods:**

A cross-sectional study was conducted using face-to-face interviews with women, routinely attending health centers, who met the inclusion criteria. Descriptive analysis was undertaken to report data.

**Results:**

A total of 324 women were included in the study. Results revealed low awareness about cervical cancer (19.6 %) and a very high acceptability of VIA screening (94.5 %). Of the 306 women screened, 99 % stated that they would recommend the VIA testing to their friends and female relatives. All those women who were screened negative expressed their intent to repeat the test every three years. Those found VIA positive affirmed they would perform confirmatory explorations. The majority (96.3 %) of the women believed that screening by VIA could save their lives; cervical cancer was a concern for 98.6 %; and only 11.6 % felt anxious about repeating the VIA test. The majority of women (98.6 %) were satisfied with the service received at the health center.

**Conclusions:**

This study showed that the participants had a strong perception about cervical cancer screening and were willing to have further confirmation or future retests.

**Electronic supplementary material:**

The online version of this article (doi:10.1186/s12905-015-0268-0) contains supplementary material, which is available to authorized users.

## Background

Cervical cancer is a major public health problem in Morocco. It is the second most common cancer in women after breast cancer [1]. It is estimated that there are 2258 new cases per year (14.3/100 000P-Yrs) [1, 2], diagnosed at very late stages, therefore, delaying their therapeutic care and complicating their cure; 2/3 of reported cases of cervical cancer are diagnosed and managed at a very advanced stage [3].

To cope with this problem, Morocco is progressively undertaking a screening program based on visual inspection of the cervix with acetic acid (VIA), used as a screening tool in primary health centers, by trained doctors, midwives, and nurses. The eligible women are those aged between 30 and 49. Colposcopy and/or directed biopsy were performed on VIA-positive women in newly built specialized centers named reference centers for reproductive health. Treatment by loop electrosurgical excision procedure (LEEP) was offered to those with cervical intraepithelial neoplasia (CIN) [4]. This program was initiated towards the end of 2010-early 2011 in only two regions in the country and scaled up to 7 regions by 2014.

Meknes-Tafilalet Region is one of the first areas where the program was implemented in Morocco. It consists of six provinces: Meknes, El Hajeb, Ifrane, Khénifra, Midelt and Errachidia. Early detection of cervical cancer is available in 122 health centers. Three reference centers were built and equipped to provide colposcopy, biopsy and LEEP. The region has had a Regional Oncology Center since June 2014. Before this date, women diagnosed positive were referred to the University Hospital Center of Fez or National Institute of Oncology in Rabat.

A pilot study revealed a low compliance rate to screening for cervical cancer; only 6 % of the women in the target age (30 to 49 years old) was screened, a high rate of colposcopy referral due to positive VIA test (70 %) and a low treatment rate of CIN by LEEP (18 %) [5].

Morocco’s program was the subject of an assessment of the progress made in the implementation of commitments to the tripartite partnership agreement between the Health Ministry, Lalla Salma Foundation for the Prevention and Treatment of Cancers, and support from UNFPA, the United Nations Population Fund in five regions of Morocco [6], but no study had been conducted to explore the perception of the program from eligible women and their satisfaction with regard to the services offered in this program. The main goal of the study reported in this paper is to identify the perception and satisfaction of cervical cancer screening via Visual Inspection by Acetic acid (VIA) among eligible women in the Meknes-Tafilalet Region, Morocco.

## Methods

We chose cross-sectional descriptive study during February 2014 using a sample of 24 health centers, which represent 20 % of total health centers that perform VIA screening in the region (Additional files 1 and 2). These were selected using a simple random sample proportional to the number of health centers in urban and rural areas in each of the 6 provinces of the region. This study was conducted on women routinely attending health centers who met the inclusion criteria, i.e., from the Meknes-Tafilalet Region, especially those living in the geographical area of the selected health centers; aged between 30 and 49 years; and recognized as being sexually active. Exclusion criteria were women who had been previously treated for cervical cancer; those pregnant beyond 8 weeks; those who were up to 6 weeks postpartum; and women with psychological and/or mental problems. The recruitment of women was done one day per health center.

In each province, one female interviewer from another province was selected to collect data, so the women could express their true feeling and give their views without hesitation. The interviewer recruited women when they were leaving the health centers and interviews took an average of 12 min. Screened women and those who refused to join the program were interviewed by trained female interviewers. The questionnaire was based upon those used in similar studies [7–9], and consisted of 6 sections: (i) social and demographic data, (ii) perception of the cervical cancer screening program, (iii) satisfaction regarding various aspects of the screening service offered at the centers, (iv) global satisfaction, (v) difficulties in accessing a VIA test (vi) reasons for rejecting screening. Some questions were assessed by choosing yes or no; others were measured using the Likert scales (Additional file 3). This questionnaire was originally written in French (the second Moroccan state language), but was conducted in the local dialect. Understanding and validation of the questionnaire was assured by giving training to investigators and a pilot study in health centers excluded from this study.

For each woman, confidentiality and free choice whether to participate in the interview were explained. Women were included in this study when they consented verbally given the high rates of illiteracy among the female Moroccan population. The protocol and the instruments used were approved by the ethics committee of Fez University Hospital Center. The ethics committee approved obtaining verbal consent only as there was potential for less than minimal harm to the participants of the survey. The data concerned the social-demographic characteristics (age, level of education, health insurance, residence of patients, etc.), knowledge of women (cervical cancer, the cervical screening program, etc.), perception and satisfaction of cervical cancer screening. Data collected from women was entered and analyzed using Epi Info 2000.

For quantitative data, descriptive analysis was conducted to define socio-demographic characteristics, awareness, perception and satisfaction of cervical cancer screening. Categorical variables will be summarized by frequencies and proportions; and continuous variables will be summarized by means and standard deviations.

## Results

### Demographic data for the women interviewed

Atotal of 324 women participated in the study was. Only 18 women refused to be screened. The mean age was 39.2 ± 5.7. Approximately 60 % of participants were aged between 30 and 40 years and nearly two-thirds had fewer than three children. Most of the women were married (87.6 %). Almost two-thirds were illiterate, 60 % were from rural areas and 90 % were housewives. Nearly half of the women reported that the total household incomewas lower than the minimum income, and one third stated that they did not have medical insurance (see Table 1).

**Table 1:** Demographic data of the women interviewed

Characteristics (*n* = 324)^a^	Distribution
Age (Mean ± SD)	39.2 ± 5.7
[30–35]	94 (29)
[35–40]	94 (29)
[40–45]	77 (23.8)
[45–50]	52 (16)
Parity	
[0–3]	211 (65.1)
[3–6]	100 (30.8)
≥6	13 (4)
Origin	
Urban	129 (39.8)
Rural	195 (60.1)
Marital Status	
Never Married	8 (2.5)
Married	284 (87.6)
Divorced	20 (6.2)
Widowed	10 (3)
Separated	2 (0.6)
Health insurance	
CNOPS	35 (10.8)
CNSS	33 (10.2)
RAMED	148 (45.7)
No insurance	108 (33.3)
Education	
Illiterate	202 (63.3)
Primary	74 (23.2)
Secondary	28 (8.7)
College	9 (2.8)
Others (literacy classes, Koranic school…)	6 (1.8)
Occupation	
Housewife	290 (90)
Maid	21 (6.5)
Professor	1 (0.3)
Others (manuel worker, saleswoman, seamstress, artisan…)	9 (2.7)
Monthly Family Income ($)	
<267	156 (48.3)
267–581	61 (18.9)
581–1163	23 (7.12)
>1163	42 (13)
Not known	41 (12.7)

^a^n varies between items because of missing data

### Knowledge of women about cervical cancer and the screening program using VIA

The participants’ knowledge of cervical cancer was limited; more than a half (57.7 %) of the women who attended health centers did not know the causes of cervical cancer, 51.5 % were unawareof the link between persistence of HPV infection and cervical cancer. Even the symptoms of cervical cancer were previously unheard of for 45.3 % of the women. Only 19.6 % of the women showed high levels of awareness with respect to cervical cancer, especially the mode of transmission, the main cause, and the symptoms.

A sizable percentage of women had heard of the cervical cancer program (84.5 %). Only 15.5 % had no prior awareness of this program. Of those who knew about the program, 85.4 % had the information from television. However, further enquiry revealed that 69.3 % of the women had very little knowledge about the necessity and mode of cervical cancer screening. This highlights the gap in the education program being broadcast in the television.

### Perception of the screening program from the perspective of the women interviewed

Cervical cancer was a concern for 98.6 % of the women. Only 11.6 % of them said they felt anxious about repeating the VIA test. Fear of having cervical cancer was the leading cause of anxiety felt by women (90 %). All women who screened negative expressed their intention to repeat the test every three years.Those found VIA-positive affirmed they would perform confirmatory explorations. The majority, 96.3 %, believed that screening could save their lives, and the fact that the test was free of charge encouraged almost all women to participate in the program (99.7 %). Virtually all women interviewed (99 %) said they would recommend the VIA test to their friends and female relatives. The majority (96.2 %) confirmed that the doctor/nurse at the health center encouraged them to repeat testing regularly (see Table 2).

**Table 2:** Perception of the screening program from the perspective of the women interviewed

Perception items (*n* = 306^a^)	Strongly agree	Agree
Cervical cancer is a disease that worries me	275 (85.6)	42 (13)
Screening for cervical cancer repeated every three years could save my life	282 (87.6)	28 (8.7)
Free testing encourages me to participate in the program	310 (96.3)	11(3.4)
The doctor/nurse of the center believed that the regularity of VIA testing was important	254 (79.1)	55 (17.1)
Recommendation of the test to relatives and friends	286 (98.3)	2 (0.7)
If the test was negative, repeat screening test for cervical cancer every three years (*n* = 264)	263 (99.6)	1 (0.4)
If the test was positive, intention to have further tests (*n* = 40)	38 (95)	2 (5)

^a^is variable with missing data

The mean age of those who declined (18) to join the program was similar to that of the entire cohort (324) of eligible women (39 ± 8). Eight women reported that they were menstruating; two women did not stat any reasons; for the remaining non participants, the reasons included lack husband’s permission, lack of time, financial restraints or having another commitment.

Among the 306 women who were screened, 26.5 % complained of some discomfort during VIA test procedures. One in seven women stated the insertion of the speculum caused pain and discomfort and one in eight reported having a burning sensation after the application of acetic acid. One out of ten women shared their fear of having an abnormal result. External conditions were also reported by several women; some complained of the cold felt in the examination room (one in three), others were frustrated by the long waiting time (one in nine); and the lack of privacy was referred to by one woman in eleven.

The positive aspects of the program most frequently mentioned by women were: availability of a program for early detection of cervical cancer (43.6 %), the test being free of charge (45.6 %), speedy results (18.5 %), confidence in the test (18.1 %), the proximity of the cervical cancer screening center to their homes (7.4 %), the quality of reception (7.7 %), continuity of care (5.4 %), and increased awareness of women about the disease (5.0 %).

### Satisfaction levels among women attending the cervical cancer screening program

Regarding overall satisfaction related to the service received at the health centers, most of the women (98.6 %) answered “highly satisfied” or “satisfied”. The majority of women were highly satisfied with the availability of the screening program using VIA (95.6 %), the schedules of screening proposed (84.2 %), and the timetable for the screening services at health centers (84 %). The women interviewed appreciated the skills of health personnel (doctors and nurses) (97 %) and felt satisfied with the reception they received (97.3 %), and with the duration of the visit to the health center (86 %) (See Table 3).

**Table 3:** Satisfaction levels among women attending the cervical cancer screening program

Satisfaction items (*n* = 306^a^)	High satisfied	Satisfied
Organization of program		
Availability of program in health centers	171 (56)	121 (39.6)
Screening schedule	158 (51.8)	99 (32.4)
Planning testing activities	144 (47.2)	112 (36.7)
Quality of screening experience		
The quality of the reception	187 (61.3)	110 (36)
Respect for privacy by staff	212 (69.5)	84 (27.5)
Technical competence	169 (55.4)	127 (41.6)
Duration of the visit at the center	159 (52)	104 (34)
Interaction with medical staff		
Clarity of information	166 (54.2)	123 (40.2)
Information provided about VIA test	181 (59.3)	115 (37.7)
Use intelligible words	164 5(53.6)	128 (41.8)
Listening time	178 (58.1)	107 (35)
Time allotted to these explanations	170 (55.9)	106 (34.8)
Calming the fears of women	170 (55.5)	128 (41.8)
Facility characteristics		
Privacy level of the examination room	242 (79)	62 (20,2)
Cleanliness of examination room and equipment	164 (53.6)	122 (39.8)
Cleanliness of material used	149 (48.7)	58 (18.9)
Overall satisfaction with the service received	169 (55.6)	131 (43)

^a^is variable with missing data

Concerning the interaction with medical staff, almost all study participants (94.4 %) stated that the information provided by health staff was clear. Health professionals used intelligible words in 95.4 % of cases. Regarding the VIA test procedure, the majority of women (97 %) were satisfied with the information provided, and 90.7 % with the time allotted to these explanations. There was a high level of satisfaction concerning the listening time that was given by health professionals (reported by 93.4 % of study participants), and women said they were reassured by 97 % of health staff (See Table 3).

Facility characteristics were also appreciated by study participants; almost all women (99.2 %) reported that their examination rooms offered enough privacy, and 97 % of women felt that health staff respected their privacy. The examination rooms were considered clean and adequate for 93.4 % of the women. However, only 67.6 % of the study participants felt that the materiel and equipment used was clean and 30.7 % had no opinion about this topic (See Table 3).

## Discussion

The present study reports that women participating in screening programs had a favorable attitude toward the VIA testing, despite their poor knowledge of cervical cancer. Care provided in cervical cancer screening programs was really appreciated by the target population. Positive perceptions of the screening program using VIA testing were reported by other studies conducted in various contexts [10–14]. The VIA test is a newly delivered service in health centers in Morocco, however, only few women refused to be screened (18 out of 324). To date, no national awareness campaign aimed at the target population of the program has been carried out, given that the program is not yet widespread in all regions of Morocco. The providers tried to convince those women who frequently attend health centers to have the test, or sometimes asked local NGO’s assistance to improve women’s uptake. The reason behind this high acceptability is most likely related to Moroccan’s perception of this illness. Moroccan people believe that it is a highly lethal and incurable disease even after treatment; few people believe it is possible to be cured of cancer [15]. This highly negative perception might encourage them to accept and believe in screening programs to save their lives.

The women had little or no knowledge cervical cancer. The Moroccan population is not adequately informed about cancerin general; knowledge of the diseaseis still poor and confused, particularly concerning the causes, symptoms, and available treatments [15]. In another context, knowledge about cervical cancer was poor among the women who underwent the screening program [10]. However, 84 % of women declared that they knew about the cervical cancer screening program, and 85.5 % of those got the information from television, but in fact no nationwide sustained campaign with regard to cervical cancer program was done. It could be only the indirect effect of breast cancer awareness campaigns. Usually, we noted that this type of campaign mobilized women also to screen for cervical cancer by VIA. For these reasons, women had poor information about cervical cancer and screening program.

Study participants felt very comfortable post-screening after VIA; especially those who had a negative result. They were reassured about the health of their cervix, and they were happy to have a free test with a quick result. No complex complaints were reported by study participants, similar to data previously reported in others studies [10–12, 14]. Some Screened women complained of pain experienced during the insertion of the speculum; this problem could be resolved by choosing an appropriately sized speculum for each woman and putting the women at ease during VIA test procedures. Some women reported a sensation of stinging or burning in their vagina caused by the diluted acetic acid (5 %) used for VIA, but this sensation is normally temporary and disappears within a few hours. It was surprising that Moroccan women did not report either having heard of or had vaginal discharge after a VIA test as has been demonstrated in other contexts [12]. This observation may perhaps be explained by the fact that providers respect the standards of hygiene and sterilization of instruments. However, feeling cold during the VIA test procedures was the most frequently reported complaint by women in the post-screening stage. The selected region is a mountainous area; with snow and cold temperatures during the period of data collection.

Despite the high satisfaction of Moroccan women with the quality of care provided by the screening program, they did suggest further improvements in the reception conditions (availability of waiting room, cleanliness, chairs, etc.), the availability of skilled staff, and reduction in the waiting time. The fact that women were satisfied with the services which were provided was reported by other studies carried out in others countries [12, 13, 16]. Women’s satisfaction guarantees trust and greater participation of women in the program [17].

This study was conducted as part of the evaluation of the implementation of the program in Meknes-Tafilalet Region; there was no previous feedback from screened women. The sample of women was a convenience sample, and was not representative of the whole Moroccan population despite the large sample. Only women already attending a health center (albeit for any reason) were invited to undergo cervical examination and to participate in this study. With the present study, we can expect to generalize the results with some reservations; social and cultural conditions could affect the results in the others regions of Morocco. The results will be important for regional and national policy makers to improve the program and the procedures of VIA testing. However, for the small sample of women who rejected the VIA test, it was not possible to explore the reasons in detail or to investigate the factors associated with their refusal.

## Conclusions

This study confirmed that Moroccan women had a favorable attitude toward the VIA testing program, despite their poor knowledge of cervical cancer. Care provided in cervical cancer screening programs was really appreciated by the target population. Barriers were very few compared with the benefits of the program, but efforts are required to upgrade health centers and to reduce discomfort felt by women who underwent the procedure. Awareness strategy is needed in the regions where the program was implemented to increase women’s knowledge about cervical cancer and improve their participation.

## Additional files

Additional file 1:
**STROBE Statement—Checklist of items that should be included in reports of** ***cross-sectional studies.*** (PDF 18 kb) Additional file 2:
**Health Centers included in this study.** (DOCX 13 kb) Additional file 3:
**Evaluation of early detection program for cervical cancer - Questionnaire used to interview women included in this study.** (DOC 222 kb)

## Abbreviations

VIA: Visual inspection of the cervix with acetic acid
LEEP: Loop electrosurgical excision procedure
CIN: Cervical intraepithelial neoplasia
UNFPA: United Nations Population Fund
