# Supplementary material for: Perception and satisfaction of cervical cancer screening by Visual Inspection with Acetic acid (VIA) at Meknes-Tafilalet Region, Morocco: a population-based cross-sectional study
Source: BMC Womens Health. 2015 Nov 24;15:106. doi: 10.1186/s12905-015-0268-0 (PMC4657367; doi:10.1186/s12905-015-0268-0)
Supplement: Additional file 2: — Health Centers included in this study. (DOCX 13 kb) [file 12905_2015_268_MOESM2_ESM.docx]

| **Health Centers included in this study** | | |
| --- | --- | --- |
|  |  |  |
| **Provinces** | **Health centers** | **Area** |
| **Meknes** | Marjane | urban |
|  | Riad | urban |
|  | Jbabra | urban |
|  | Diour Essalam | urban |
|  | Ain Orma | rural |
|  | Dar Oum Soltane | rural |
|  | Oum Rabiae | Urban |
|  | Ouisslane | Urban |
| **El Hajeb** | El Hajeb I | Urban |
|  | Rass Ljiry | Rural |
| **Khenifra** | Amalou II | Urban |
|  | My Bouazza | Rural |
|  | Aguelmous | Rural |
|  | Asaka | Urban |
| **Ifrane** | Tarik | Urban |
|  | Oued Ifrane | Rural |
| **Midelt** | CSU Hassan II | urban |
|  | Zaida | Rural |
|  | Boumia | Rural |
| **Er-Rachidia** | Oulad Bounaji | Urban |
|  | Abderahman Sahraoui | Urban |
|  | Ktaa Loued | Rural |
|  | Goulmima | Rural |
|  | Siffa | Rural |
