# Supplementary material for: Perception and satisfaction of cervical cancer screening by Visual Inspection with Acetic acid (VIA) at Meknes-Tafilalet Region, Morocco: a population-based cross-sectional study
Source: BMC Womens Health. 2015 Nov 24;15:106. doi: 10.1186/s12905-015-0268-0 (PMC4657367; doi:10.1186/s12905-015-0268-0)
Supplement: Additional file 3: — Evaluation of early detection program for cervical cancer - Questionnaire used to interview women included in this study. (DOC 222 kb) [file 12905_2015_268_MOESM3_ESM.doc]

**Evaluation of early detection program for cervical cancer**

**Questionnaire for women (health center)**

who received program benefits

and those who refused to join in the program

| **Province Name: ___________________________**  **Name of Health Center : _________________________**  **Rural Urban Communal**  **Number of women : /______/______/______/** | **Date of Interview: /_____/______/__2014__/**  **Day Month Year**  **Name of the interviewer : ________________________** |
| --- | --- |

| ***Instructions:***  **1. Read the questions and answers to the woman**  **2. Note the woman's responses and use "does not apply" if this is the response**  **3. Write the comments of the women accurately (use the same terms)**  **4. Remember that the woman is always free to respond or not to one or more questions**  **5. At the end of the questionnaire, check if the woman wishes to add other items**  **6. Record your comments on the course of the interview when you're alone**  **7. Make sure that you have done all the questions** | ***Hello madam .........***  ***My name is …….and I m working in ............ city…..***  ***Lalla Salma Foundation, prevention and treatment of cancer, in partnership with the Ministry of Health and Fez Medical School, launched a study which aims to assess the early detection program for cervical cancer. For this we want to know your opinion regarding the services offered in this program.***  ***I will ask you a series of questions and answers and you need only tell me the answer that best represents your opinion. There are no right or wrong answers. Anything you say will be interesting to me.***  ***If you are having difficulty understanding, please tell me about that. I might rephrase the question to make it clearer for you.***  ***I wish to inform you that your answers will remain anonymous and confidential during the drafting of the final report of the study and you are free to accept or refuse participation in this interview.***  ***I rely on your support and thank you for agreeing to conduct this interview with you.***  ***Do you agree to participate in the study?***  **1 Yes 2 No** |
| --- | --- |

**Section 1 : Test VIA**

1. What was the reason for your visit?

01 Cervical screening cancer 05 Nursing care

02 Family Planning 06 Visit information

03 Vaccine 07 Another reason:…………………………………………

04 Medical consultation

1. Have you ever performed a cervical cancer screening VIA test?

1 Yes 2 No (if yes, go to question 5)

1. Today, have you have benefited from a cervical cancer screening VIA test?

1 Yes 2 No (if not, and if the woman refused to join the program today

or before when collecting data, go to Section 4 Question 3)

1. Why did you choose this health center for cervical cancer screening?

01 Near to your workplace 05 Easy accessibility (transport, cost)

02 Near of your residence 06 Lack of cervical cancer screening service at the center nearest

to you

03 Recommended by someone 07 Confidence in staff

04 Good reputation of the center 08 Another reason:…………………………………………

| A | | In the reception upon arrival at the health center | | | | | | | |
| --- | --- | --- | --- | --- | --- | --- | --- | --- | --- |
| **What extent are you satisfied** | | | | **Very satisfied** | | **Satisfied** | **dissatisfied** | **Very dissatisfied** | **Not applicable** |
|  | | the cleanliness of the premises, the equipment and the waiting room equipment | | 4 | | 3 | 2 | 1 | 9 |
|  | | Waiting time before receiving the test | | 4 | | 3 | 2 | 1 | 9 |
|  | | the way in which the staff greeted you on arrival (*please answer Not Applicable (NA) if there were no official reception staff*) | | 4 | | 3 | 2 | 1 | 9 |
|  | | the clarity of the information provided by staff about the program (*please answer NA if there were no official reception staff*) | | 4 | | 3 | 2 | 1 | 9 |
|  | | the interest expressed by staff to your questions and your concerns  (*please answer NA if you did not have questions*) | | 4 | | 3 | 2 | 1 | 9 |
| B | VIA examination room | | | | | | | | |
| **What extent are you satisfied** | | | **Very satisfied** | | **Satisfied** | | **dissatisfied** | **very dissatisfied** | **Not applicable** |
|  | the cleanliness of the premises and the examination room equipment | | 4 | | 3 | | 2 | 1 | 9 |
|  | how the staff let you make your decisions to join or not to the program | | 4 | | 3 | | 2 | 1 | 9 |
|  | The discretion of the staff about you (*not talking to anyone about your problems*) | | 4 | | 3 | | 2 | 1 | 9 |
|  | the time taken by the nurse/ doctor to listen to you | | 4 | | 3 | | 2 | 1 | 9 |
|  | the time allocated to explain the stages of the test and make you feel confident | | 4 | | 3 | | 2 | 1 | 9 |
|  | the degree of intimacy given to you in the examination room | | 4 | | 3 | | 2 | 1 | 9 |
|  | the respect the staff gave for your privacy (closing the door, knocking on the door before entering the examination room, use of the screen ...) | | 4 | | 3 | | 2 | 1 | 9 |
|  | the safety of the equipment used in the examination (clean and sterile equipment) | | 4 | | 3 | | 2 | 1 | 9 |

| C | The information given to you | | | | | |
| --- | --- | --- | --- | --- | --- | --- |
| **What extent are you satisfied** | | **Very satisfied** | **Satisfied** | **dissatisfied** | **very dissatisfied** | **Not applicable** |
|  | efforts by staff to explain the program and VIA test, with intelligible words | 4 | 3 | 2 | 1 | 9 |
|  | the efforts made by staff to ease your worries | 4 | 3 | 2 | 1 | 9 |
|  | the information received on the test result | 4 | 3 | 2 | 1 | 9 |
|  | the clarity of the information received, generally | 4 | 3 | 2 | 1 | 9 |

**Section 2 : General satisfaction**

| D | Overall satisfaction Elements | | | | | |
| --- | --- | --- | --- | --- | --- | --- |
| **What extent are you satisfied** | | **Very satisfied** | **Satisfied** | **dissatisfied** | **very dissatisfied** | **Not applicable** |
|  | the availability of a cervical cancer screening service at the your health center | 4 | 3 | 2 | 1 | 9 |
|  | screening schedule proposed by the health center | 4 | 3 | 2 | 1 | 9 |
|  | the planning of cervical cancer screening activities at your health center | 4 | 3 | 2 | 1 | 9 |
|  | the competence of doctors | 4 | 3 | 2 | 1 | 9 |
|  | the competence of nurses | 4 | 3 | 2 | 1 | 9 |
|  | the duration of the visit to the health center | 4 | 3 | 2 | 1 | 9 |
|  | Courteous and friendly staff | 4 | 3 | 2 | 1 | 9 |
|  | overall, how did you find the services received during the procedure? | 4 | 3 | 2 | 1 | 9 |

1. **Do you have any recommendations to improve the reception and screening services at your health center?** 1 Yes 2 No If yes, which?

|  |  |  |  |  |
| --- | --- | --- | --- | --- |
|  |  |  |  |  |
|  |  |  |  |  |
|  |  |  |  |  |
|  |  |  |  |  |
|  |  |  |  |  |
|  |  |  |  |  |
|  |  |  |  |  |

**Section 3 : Care accessibility problems**

1. **What do you think about the cost of**

**your cervical cancer screening consultation?** 1 **C**orrect

2 High

3 Very high

4 I didn’t have expenses

5 No comment

6 It's not me who paid

9 I don’t know

1. **On average, how many hours did you need to get to the center?**  /______/______/

hour min

1. **What means of transportation did you use**

**to get to the health center?**  1 Walking

2 Bus

3 Taxi

4 Own way: *car, bicycle, tractor, motorbike…*

5 Other

1. **Did you find particular problems during your VIA procedures?**

1 Yes 2 No If yes, which?

|  |
| --- |
|  |
|  |
|  |
|  |
|  |
|  |
|  |
|  |
|  |

**Section 4: Perception of early detection program for cervical cancer**

1. **Have you ever heard of the program** 1 Yes, often

**of early detection of cervical cancer?** 2 Yes, infrequently

3 No, not at all

**(if no, go to question 38)**

1. **How did you hear about the program?**

01 Television 05 Relatives (family, friends, neighbors…)

02 Newspapers 06 Associations

03 Health personnel 07 Posters

04 Radio 08 Another way - specify:…………………………………………………

1. **Did you think you have been well informed about the program?**
2. Not at all 4 Enough
3. A little 5 Very well
4. More or less

| **Did you know that** | | **Yes** | **No** |
| --- | --- | --- | --- |
|  | Cervical cancer is a disease that it is transmitted sexually | 1 | 2 |
|  | The persistence of HPV infection is the main cause of cervical cancer | 1 | 2 |
|  | Cervical cancer symptoms are: discharge, repeated infection, post coital bleeding and pain | 1 | 2 |
|  | The cervical cancer screening test is free at your health center? | 1 | 2 |
|  | The targets of the program are sexually active women, aged between 30 and 49 years? | 1 | 2 |
|  | Further tests are available and free at the reference centers for reproductive health | 1 | 2 |

What extent do you agree or disagree with the following statements

|  | **Perception items** | **Strongly agree** | **Agree** | **Disagree** | **Totally disagree** | **Not applicable** |
| --- | --- | --- | --- | --- | --- | --- |
|  | Cervical cancer is a disease that worries me | 4 | 3 | 2 | 1 | 9 |
|  | I think that cervical cancer screening every three years could save my life | 4 | 3 | 2 | 1 | 9 |
|  | Having the free test encourages me to participate in the program | 4 | 3 | 2 | 1 | 9 |
|  | For the doctor / nurse of the center, it is important that I take the VAI test regularly | 4 | 3 | 2 | 1 | 9 |
|  | **If the woman refused VAI test, go to Section 5 / Question 55** | | | | | |
| **NB** | **If the woman has performed the test, get the test results:** | Negative | Positive |  |  |  |
|  |  |  |  |  |  |  |
|  | **If the test was negative:**  I intend to repeat the cervical cancer screening test every 3 years | 4 | 3 | 2 | 1 | 9 |
|  | **If the test was positive:**  I intend to perform confirmatory explorations if necessary | 4 | 3 | 2 | 1 | 9 |
|  | I think I will recommend this test to my family and my friends | 4 | 3 | 2 | 1 | 9 |

**If the test was positive, go to Question 53**

1. **Are you anxious about retesting?** 1 Not at all 2 A little

3 More or less

4 Quite a lot

5 A lot

1. **If you are anxious about repeating the test every 3 years, please indicate why?**

|  |  |  |  |  |
| --- | --- | --- | --- | --- |
|  |  |  |  |  |
|  |  |  |  |  |
|  |  |  |  |  |
|  |  |  |  |  |

**If the woman has joined the program, go to Section 6 / Question 80**

**Section 5: The reasons for refusal**

|  | **I did not receive screening services because** | **Yes** | **No** | **Don’t know** |
| --- | --- | --- | --- | --- |
|  | The one offering me the test is a male doctor | 1 | 2 | 9 |
|  | Providers are not competent in this center | 1 | 2 | 9 |
|  | Staff are unfriendly | 1 | 2 | 9 |
|  | I want to do it in the private sector | 1 | 2 | 9 |
|  | A skilled provider is not available | 1 | 2 | 9 |
|  | The health center is very far | 1 | 2 | 9 |
|  | I have a problem of transport | 1 | 2 | 9 |
|  | I haven’t got any money. | 1 | 2 | 9 |
|  | I have to ask permission from my husband | 1 | 2 | 9 |
|  | Cervical cancer is incurable, screening is useless | 1 | 2 | 9 |
|  | The test is tedious and painful | 1 | 2 | 9 |
|  | The test is not reliable | 1 | 2 | 9 |
|  | I feel uncomfortable during the test | 1 | 2 | 9 |
|  | Lack of privacy | 1 | 2 | 9 |
|  | I have already done a test and that's enough | 1 | 2 | 9 |
|  | I'm in a hurry, it takes a lot of time to wait | 1 | 2 | 9 |
|  | I have my period | 1 | 2 | 9 |
|  | I'm still young | 1 | 2 | 9 |
|  | I have other worries at home | 1 | 2 | 9 |
|  | I have other health problems | 1 | 2 | 9 |
|  | I'm healthy, I'm not likely to get cancer | 1 | 2 | 9 |
|  | I’m afraid that cancer will be discovered | 1 | 2 | 9 |
|  | I came to the center for another reason | 1 | 2 | 9 |
|  | I made an appointment | 1 | 2 | 9 |
|  | Other - please specify | 1 | 2 | 9 |

**Section 6: Socio-demographic characteristics of women**

|  | **Characteristics** |  |
| --- | --- | --- |
|  | Age**(years)** |  |
|  | Parity |  |
|  | Profession |  |
|  | Education *(****1****: Illiterate;* ***2****: Primary ;* ***3****: Secondary ;* ***4****: College ;*  ***5****: other specify ;* **9 :** Dont know *)* |  |
|  | Origin *(****1****: Urban ;* ***2****: Rural)* |  |
|  | Marital Status *(****1****: Never Married;* ***2****: Married ;* ***3****: Divorced;* ***4****: Widowed;* ***5*** : *Separated ;* ***6****:* *Cohabit)* |  |
|  | Health insurance *(****1****: CNOPS ;* ***2****: CNSS ;* ***3****: RAMED ; 4 : No insurance)* |  |
|  | Monthly Family Income ($) (**1** : **<** 267; **2** : 267-581;  **3 :** 581-1163**; 4 :** > 1163; **5 :** Don’t know) |  |

**Thank you very much for your collaboration**
